# The Hippo/YAP signaling pathway: the driver of cancer metastasis

**DOI:** 10.20892/j.issn.2095-3941.2023.0164

**Published:** 2023-07-11

**Authors:** Tianxing Zhou, Xueyang Li, Jing Liu, Jihui Hao

**Affiliations:** 1 Department of Pancreatic Cancer, Tianjin Medical University Cancer Institute and Hospital, National Clinical Research Center for Cancer, Key Laboratory of Cancer Prevention and Therapy, Tianjin 300060, China; 2 Department of Breast Oncoplastic Surgery, Tianjin Medical University Cancer Institute and Hospital, National Clinical Research Center for Cancer, Key Laboratory of Cancer Prevention and Therapy, Tianjin’s Clinical Research Center for Cancer, Key Laboratory of Breast Cancer Prevention and Therapy, Tianjin Medical University, Ministry of Education, Tianjin 300060, China

Despite major improvements in cancer survival, metastasis is responsible for almost 90% of cancer-related mortality^1,2^. Nonetheless, the pathogenesis and molecular mechanisms of cancer metastasis remain poorly understood. Metastasis is the culmination of a complicated series of events called the metastatic cascade. During metastatic dissemination, a cancer cell from the primary tumor region (1) undergoes epithelial-to-mesenchymal transition (EMT) and adopts stem-like properties; (2) locally invades the basal membrane and the surrounding matrix; (3) induces angiogenesis and lymphangiogenesis, thus creating migration routes; (4) migrates and enters the microvasculature of the lymph and blood systems (intravasation), and becomes a circulating tumor cell (CTC); (5) survives and translocates through the bloodstream to the micro-vessels of distant tissues; (6) exits from the bloodstream (extravasation); (7) reprograms itself through mesenchymal-to-epithelial transition, and seeds and survives in the microenvironment of foreign tissues (metastatic niche); and (8) finally adapts to the foreign tissue microenvironment and forms a secondary tumor (colonization and adaption) (**Figure 1**). Furthermore, cancer cells can remodel the landscape of the tumor microenvironment (TME) and evade immune surveillance in primary and metastatic foci, thereby facilitating cancer growth and metastasis. Recently, the Hippo signaling pathway has been characterized as the key regulator mediating cancer metastasis. Thus, investigation of the mechanism underlying Hippo-mediated cancer metastasis is an emerging area of research. Targeting the Hippo signaling pathway may have promise in the treatment of metastatic cancers.

**Figure 1:**
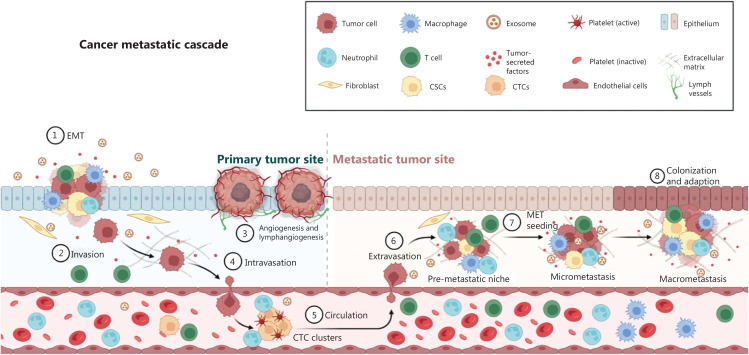
Schematic illustration of the metastasis cascade. During metastatic dissemination, a cancer cell from the primary tumor region (1) undergoes epithelial-to-mesenchymal transition (EMT) and adopts stem-like properties; (2) locally invades the basal membrane and the surrounding matrix; (3) induces angiogenesis and lymphangiogenesis, thus creating migration shortcuts; (4) migrates and enters the microvasculature of the lymph and blood systems (intravasation) and becomes a circulating tumor cell (CTC); (5) survives and translocates through the bloodstream to the micro-vessels of distant tissues; (6) exits from the bloodstream (extravasation); (7) reprograms itself through mesenchymal-to-epithelial transition, and seeds and survives in the microenvironment of foreign tissues (metastatic niche); and (8) finally adapts to the foreign tissue microenvironment and forms a secondary tumor (colonization and adaption). Furthermore, cancer cells can remodel the landscape of the tumor microenvironment (TME) and evade immune surveillance in primary and metastatic foci, thus facilitating cancer growth and metastasis.

## Overview of the Hippo/YAP signaling pathway

### The canonical Hippo/YAP signaling pathway in mammals

The Hippo pathway, originally identified in the *Drosophila* genus, is a highly conserved kinase cascade that regulates organ size^3^. The core components of Hippo pathways in mammals include MST1/2, Sav1, LATS1/2, and MOB1. In mammals, phosphorylation of MST1/2 and LATS1/2 inactivates the transcriptional co-activator Yes-associated protein (YAP) as well as its paralog, transcriptional co-activator with PDZ-binding motif. Phosphorylation of YAP or TAZ by LATS1/2 results in their interaction with 14-3-3 proteins, their cytoplasmic retention and degradation, and a subsequent decrease in the expression of their downstream gene targets. When YAP and TAZ are not phosphorylated, they accumulate in the nucleus and modulate downstream gene expression. MST1/2 are activated by Sav1, which interacts with MST1/2 through SARCH domains. MOB1, which forms a complex with LATS1/2, is phosphorylated by MST1/2, thus enhancing LATS1/2-MOB1 interaction. Moreover, YAP/TAZ interact with other proteins, such as TEA-domain (TEAD) family members and the Smad family member p63^4^.

### Regulation of the activity of the Hippo/YAP signaling pathway

In the canonical Hippo pathway, phosphorylation and dephosphorylation processes are responsible for YAP/TAZ activity in mammals^5^. The commonly phosphorylated sites in YAP/TAZ include Ser61, Ser109, Ser127 (Ser89 in TAZ), Ser164, Ser381, Ser384, and Ser387. Phosphorylation of Ser127 (Ser89 in TAZ) induces YAP/TAZ interaction with 14-3-3 proteins and subsequent cytoplasmic retention, thus diminishing the transcriptional activity of YAP/TAZ. When phosphorylated by LATS1/2 at Ser 381, YAP (Ser384/387) is primed for further phosphorylation by the casein kinase I isoform δ/ε (CKI δ/ε) in a phosphodegron and eventually promotes the ubiquitination-mediated degradation of YAP, as induced by SCFβ−TRCP^5^. Similarly, many kinases have been reported to interact with and phosphorylate YAP and TAZ, including Src, Akt, and c-Abl. Furthermore, YAP/TAZ can be dephosphorylated by the catalytic subunit of protein phosphatase-1 (PP1A) and protein phosphatase-2 (PP-2A) at Ser127 (Ser89 and Ser311 in TAZ), thus inducing translocation into the nucleus and activation of target genes^6^. Additionally, the stability and content of YAP/TAZ are regulated by ubiquitination-induced degradation. SCFβ−TRCP dependent ubiquitination is a canonical YAP1/TAZ degradation mechanism that depends on YAP/TAZ phosphorylation. We have found that syndecan binding protein (SDCBP) directly interacts with the TAD domain of YAP1; inhibits casein kinase I isoform δ/ε (CKI δ/ε)-mediated YAP-Ser384/Ser387 phosphorylation; and subsequently prevents YAP from undergoing β−TRCP-mediated proteasomal degradation^7^. Other E3 ligases (for example, FBXW7 and NOT4) and deubiquitinases (for example, USP9X) also increased the stability of YAP/TAZ in a ubiquitination-dependent manner. Beyond phosphorylation and ubiquitination, O-GlcNAcylation is also involved in the regulation of YAP/TAZ^8^. O-GlcNAc transferase (OGT) directly induces O-GlcNAcylation at YAP1 Ser109 and Thr241, and consequently interferes with the interaction between YAP and LATS1/2 and suppressing the phosphorylation of YAP. Studies are increasingly indicating that the localization and activity of YAP are regulated by liquid-liquid phase separation^9^. Specifically, hyperosmotic stress-induced condensation of YAP into liquid droplets shifts YAP’s distribution from the cytoplasm to the nucleus. Subsequently, YAP drives the transcription of genes involved in cell proliferation. Together, the above mechanisms indicate potential Hippo/YAP pathway targets.

## Mechanisms of Hippo/YAP signaling pathway-mediated cancer cell metastasis

### The Hippo/YAP signaling pathway and (CSCs)

**Figure 2:**
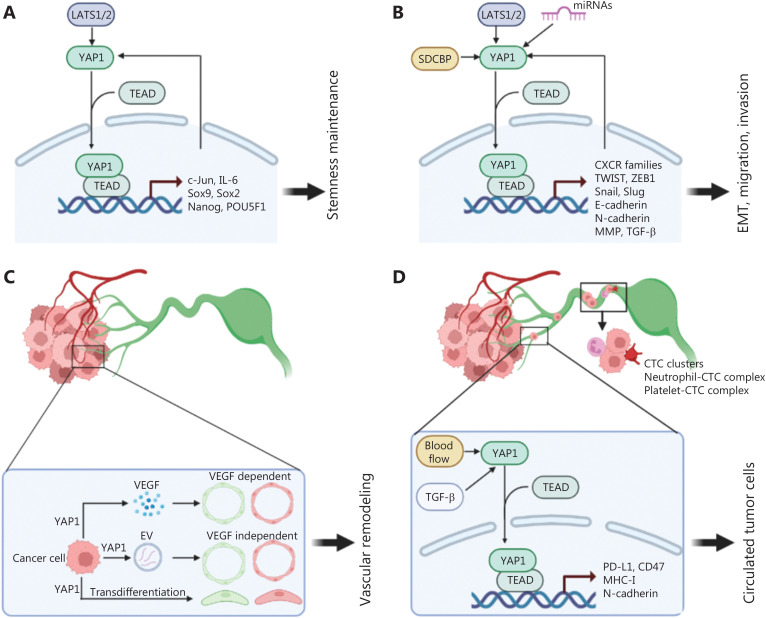
Mechanism of the Hippo/YAP1 signaling pathway in regulating cancer metastasis. (A) Roles of Hippo/YAP1 in tumor initiation. The YAP1/TEAD complex drives tumor initiation *via* inducing the expression of stemness-associated factors, which subsequently interact with the Hippo/YAP1 pathway in a positive-feedback loop. (B) Roles of Hippo/YAP1 in tumor epithelial-mesenchymal transition and motility. The YAP1/TEAD complex induces the expression of EMT/motility-associated factors, which subsequently interact with the Hippo/YAP1 pathway in a positive-feedback loop. Other regulators such as SDCBP and miRNAs also influence the activity of Hippo/YAP1. (C) Roles of Hippo/YAP1 in vasculature remodeling. Hippo/YAP1 regulate angiogenesis and lymphangiogenesis in a VEGF-dependent/independent manner. Furthermore, Hippo/YAP1 may participate in the transdifferentiation of cancer cells into endothelial cells. (D) Roles of Hippo/YAP1 in circulating tumor cells. Hippo/YAP1 can result in dysfunction of tumoral PD-L1, CD47, MHC-I, etc., thereby inducing tumoral immune evasion in the circulation. Platelets and neutrophils adhere to the surfaces of CTCs and facilitate CTC motility.

Substantial evidence indicates that a small but distinct subset of tumor cells can multi-differentiate, self-renew, and drive tumor formation. These tumor cells are therefore called CSCs. CSCs detach from primary tumor sites and migrate to distant organs or lymph nodes, then form metastatic foci. The gene expression profile in CSC-enriched tissues of breast cancer highly overlaps with that of YAP/TAZ-driven target genes, thus strongly suggesting the importance of YAP/TAZ in CSCs^10^. Furthermore, the role of YAP/TAZ in CSC maintenance has also been confirmed in cancers including pancreatic cancer, ovarian cancer, HCC, and glioblastoma. Mechanistically, YAP/TAZ regulate cellular stemness by inducing the expression of canonical stemness-associated genes, such as SOX2, SOX9, NANOG, and POU5F1. We have found that YAP/TEAD transcriptionally induce the expression of c-Jun, thereby regulating the expression of stemness-associated genes in PDAC^11^. Additionally, other stemness-associated proteins, including Sox2, Snail, TWIST, and IL-6, interact with the Hippo/YAP signaling pathway and consequently regulate CSCs (**Figure 2A**).

### The Hippo/YAP1 signaling pathway and EMT

The initial stages of the metastatic cascade require epithelial cancer cells in the primary region to lose their polarity and cell-cell adhesion, and promote transdifferentiation into a mesenchymal phenotype in the EMT process. The positive correlation between the YAP/TAZ and EMT signatures has also been observed in multiple tumor types. Mechanistically, YAP/TAZ promote cancer EMT through inducing critical EMT-orchestrating transcriptional factors including TWIST, ZEB1, and snail family zinc finger 1/2 (Snail and Slug), which control the expression of EMT-associated gene profiles, such as those of E-cadherin, N-cadherin, vimentin, and matrix metalloproteinases. We have found that SDCBP increases the stability and content of YAP, thereby modulating the expression of E-cadherin, N-cadherin, and vimentin, and further promoting EMT in pancreatic cancer^7^. Furthermore, EMT driven by TGF-beta, neurofibromin-2, SnoN, and PAR1 is also determined by the nuclear accumulation of YAP/TAZ. Therefore, YAP/TAZ initiate the earliest event in the cancer metastatic cascade by directly or facilitating cellular EMT (**Figure 2B**).

### The Hippo/YAP1 signaling pathway, and cellular migration and invasion

Dysregulation of the Hippo signaling pathway has been found to control cancer migration and invasion. The abnormal expression of MST1/2 and LATS1/2, direct regulators of YAP/TAZ, also affects these 2 processes in cervical cancer and gastric cancer. Moreover, other upstream regulators (such as miRNAs and RAR PTEN) modulate cellular migration and invasion *via* YAP/TAZ. Mechanistically, the dynamic rearrangement of the cytoskeleton is responsible for cellular motility. Notably, LATS1, an actin binding protein, suppresses actin polymerization, thus further decreasing cellular spreading and migration. The rearrangement of the cytoskeleton facilitates the formation of invadopodia, which release many proteases (such as MMP family members) and further induce matrix degradation. YAP1 promotes TEAD4 binding to the enhancer region of TIAM1, thereby activating TIAM1 expression, increasing RAC1 activity, and subsequently inducing invadopodia formation and metastasis in breast cancer^12^. Notably, our recent study has indicated that SDCBP increases invadopodia formation in PDAC in a YAP1-dependent manner^7^. Moreover, Hippo/YAP control the expression of chemokine receptors (such as the CXCR4, CXCR5, CXCR7, and CCR families) in cancer cells and consequently promote the migration of these cells to metastatic sites through receptor-ligand interactions. Beyond soluble signals, cells sense their microenvironment through mechanical cues that are derived from the extracellular matrix and regulate the Hippo signaling pathway.

Most invasive solid tumors predominantly display collective invasion rather than invasion by individual cells: groups of cells invade the peritumoral stroma while maintaining cell-cell contacts. The cells remain cohesive by expressing cell–cell junction molecules such as cadherins or adhesion receptors in the immunoglobulin superfamily. Notably, YAP phosphorylation and inactivation are responsible for collective migration of *Drosophila* and cancer^13^. Therefore, dissecting the roles of Hippo/YAP in cancer migration and invasion, particularly in collective motility, may eventually reveal novel targets for therapeutic applications against metastatic cancer (**Figure 2B**).

### The Hippo/YAP1 signaling pathway and vasculature remodeling

Metastasis to lymph nodes or distal organs is facilitated by vasculature remodeling induced by detached mesenchymal cancer cells; these remodeling processes include primarily angiogenesis and lymphangiogenesis, thus creating shortcuts for cancer cells to intravasate into the circulation. Recent studies have indicated that YAP/TAZ, core members of the Hippo pathway, contribute to angiogenesis in a VEGF-dependent manner. Furthermore, stress results in the transdifferentiation of cancer cells into endothelial-like cells, in a process called tumoral vascular mimicry. Bora-Singhal et al.^14^ have found that depletion of YAP1 suppresses self-renewal and vascular mimicry of stem-like cells in NSCLC. However, the role of Hippo/YAP1 in lymphangiogenesis remains controversial. One study examining the zebrafish trunk has suggested that YAP1 is indispensable for VEGFC-induced proliferation of lymphatic progenitors and lymphatic development^15^, whereas another study in a mouse model has indicated that LEC-specific YAP/TAZ restrict lymphatic sprouting with profoundly downregulated Prox1^16^. Therefore, the role of the Hippo signaling pathway in vasculature remodeling must be further investigated (**Figure 2C**).

### The Hippo/YAP1 signaling pathway and circulating tumor cells

Cancer cells disseminate from tumors by invading blood and lymphatic vessels; these cancer cells in the blood circulation, referred to as CTCs, are a focus of intense research. Sheer stress of blood flow and soluble factors (such as TGF-β) regulate the activity of YAP/TAZ^17^, thus modulating EMT properties and self-renewal capability. Furthermore, CTCs can escape immune surveillance through downregulation of the expression of major histocompatibility complex and immune checkpoint molecules (such as PDL1, HLAE, and CD47) or through support from platelets and neutrophils, which are regulated by the Hippo/YAP1 pathway. Interestingly, recent studies have indicated that neutrophils and platelets escort CTCs and consequently enable tumor progression. Furthermore, high proportions of CTC clusters (often referred to as collective cancer cells), rather than single CTCs, are often observed in several metastatic tumor types. The biological behavior of CTCs remains unclear, and whether the Hippo/YAP1 pathway participates in these events is worthy of exploration (**Figure 2D**).

### The Hippo/YAP1 signaling pathway and colonization-adaptation of disseminated cells at metastatic foci

CTCs undergo mesenchymal-to-epithelial transition, exit from the bloodstream (in a process referred to as extravasation), seed and survive in the microenvironment of distant tissues, and finally adapt to the foreign microenvironment and form metastatic foci. Before seeding in foreign organs, cancer cells normally secrete several inflammatory factors and exosomes, which induce the formation of the pre-metastatic niche^18,19^, and attract disseminating tumor cells and immune suppressive cells. However, studies on YAP regulation of exosome generation are rare. To date, only one study has indicated that YAP and its m5C modification stimulate exosome secretion in a Mycn- and SOX10-dependent manner in lung adenocarcinoma^20^. Er et al.^21^ have reported that pericyte-like spreading by disseminated cancer cells activates YAP and MRTF for metastatic colonization. Furthermore, certain lung cancers rapidly metastasize to multiple sites, whereas others, such as breast and prostate carcinomas, often develop metastatic colonies over the course of years, in a relatively limited number of sites. Nevertheless, whether the organ preference for cancer colonization depends on the Hippo signaling pathway is unclear. When adapting to foreign environments, cancer cells undergo rapidly expanding macro-metastasis. However, how metastatic cancer cells in distant organs remodel the metastatic niche remains elusive. Recently, Ombrato et al.^22^ have examined the host tissue cellular environment in regions surrounding metastatic cancer cells and found that these cancer-associated parenchymal cells exhibit stem-cell-like features. However, studies of YAP’s regulation of the metastatic niche are scarce.

### Crosstalk between the Hippo/YAP1 signaling pathway and the TME facilitates cancer metastasis

**Figure 3:**
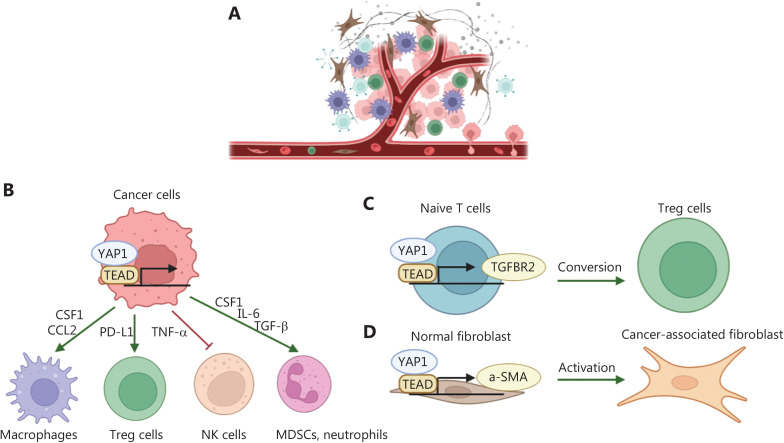
Roles of Hippo/YAP1 in tumor microenvironmental remodeling. (A) Schematic illustration of the TME. (B) The tumoral Hippo/YAP1 signaling pathway regulates the function of immune cells in a cytokine-dependent manner. (C) The Hippo/YAP1 signaling pathway converts naïve T cells into Tregs *via* regulating the expression of TGFBR2. (D) The Hippo/YAP1 signaling pathway induces the activation of normal fibroblasts *via* regulation of a-SMA expression.

Evidence has indicated that the bidirectional interactions between tumor cells and the TME are the basis for metastasis, and the Hippo pathway has a core position in this network of interactions. Tumor derived Hippo signals modulate the activity of CAF, the extracellular matrix, and the anti-tumor immune response. Tumor stromal regulate their intrinsic activities and function in a Hippo signaling-dependent manner. Furthermore, stromal signals also affect the activity of the Hippo pathway in cancer. Microenvironmental cues are crucial in all steps of the metastasis cascade (**Figure 3**).

### The Hippo/YAP1 signaling pathway and cancer metabolism reprogramming in the metastasis cascade

Recently, attention has been paid to the selective and dynamic adaptation of metastasizing cancer cell metabolism in each step of the metastatic cascade. Many metastases display different metabolic traits from those of the tumors from which they originate, thus enabling survival and growth in the new environment. Crosstalk between Hippo signaling components and metabolic pathways, including lipogenesis, glycolysis, and the mTOR pathway, has been intensively investigated. Importantly, dysregulation of the Hippo signaling pathway promotes metabolic pathway reprogramming, thereby supplying nutrients to support uncontrolled proliferation. Abnormal metabolic pathway regulation also facilitates cancer growth by inhibiting the Hippo signaling pathway. All the above results suggest that the Hippo signaling pathway acts as a metabolic sensor in dynamic cancer metabolic conditions, thus providing multifaceted opportunities for anti-cancer strategies.

### Therapeutic targeting of the Hippo/YAP signaling pathway in cancer metastasis

Given the crucial role of the Hippo/YAP1 signaling pathway in cancer metastasis, efforts in drug development are increasingly focusing on targeting this pathway. To date, the main strategies for modulation of Hippo/YAP activity have included (1) targeting YAP/TAZ nuclear shuttling and expression (such as with verteporfin, A35, dichloroacetate, Dasatinib, and CA3); (2) targeting the YAP/TAZ-TEAD interaction (such as with peptides and small molecules); and (3) targeting the YAP/TAZ-associated transcriptional machinery (such as with JQ1, HDAC inhibitors, and AP1 inhibitors). Furthermore, targeting upstream regulators has provided an alternative approach. For example, we have performed high throughput drug screening of FDA-approved drugs and found that zinc pyrithione significantly down-regulates the expression of SDCBP, decreases Hippo/YAP1 pathway activity, and overcomes cancer metastasis^7^. Moreover, our screen has identified irbesartan, which inhibits the translocation of YAP into the nucleus *via* the AT1R pathway^11^. In addition, because of the crucial role of phase separation of YAP/TAZ in Hippo activity, strategies to interfere with this event should open new avenues in cancer treatment. Indeed, the recently reported anti-tumor effects of these compounds are likely not to be YAP/TAZ-specific. However, the permeability, specificity, efficacy, and safety of all these inhibitors remain to be further determined.

## Conclusions and perspectives

The Hippo/YAP signaling pathway is considered the trader of cancer metastasis. Development of feasible approaches to monitor and target the Hippo/YAP pathway in cancer metastasis remains urgently needed. The roles of Hippo/YAP in CTCs, colonization-adaptation in metastatic foci, metabolic reprogramming, and cross-talk with the TME are outstanding questions remaining to be investigated. Although only several regulators and Hippo-targeting drugs have been developed, ongoing research is increasingly identifying potential novel Hippo/YAP mechanisms in multiple cancers. More novel findings are expected to provide a roadmap for developing new therapeutic strategies targeting the Hippo/YAP pathway, thus benefitting patients with metastatic cancers.
